# Evaluation of prognostic biomarkers for exacerbation frequency in patients with chronic obstructive pulmonary disease

**DOI:** 10.1590/1806-9282.20251626

**Published:** 2026-06-19

**Authors:** Gulhan Ayhan Albayrak

**Affiliations:** 1 Health Sciences University, Şişli Hamidiye Etfal Training and Research Hospital, Department of Chest Diseases – Istanbul, Turkey.

**Keywords:** Chronic obstructive pulmonary disease, Inflammation, Exacerbations, Hospitalization, Prognosis, Diagnosis

## Abstract

**OBJECTIVE:**

Systemic inflammation is closely associated with acute exacerbations of chronic obstructive pulmonary disease and may reflect the risk of future exacerbations. Blood-based inflammatory biomarkers represent simple and practical tools for identifying patients at increased risk. The aim of this study was to evaluate the association between systemic inflammatory biomarkers measured during stable chronic obstructive pulmonary disease and overall exacerbation burden across Global Initiative for Chronic Obstructive Lung Disease stages.

**METHODS:**

This retrospective cohort study included 132 patients with chronic obstructive pulmonary disease followed for at least 2 years. Clinical, functional, and laboratory data obtained during clinically stable periods were analyzed. Systemic inflammatory indices (neutrophil-to-lymphocyte ratio, platelet-to-lymphocyte ratio, systemic immune-inflammation index, systemic inflammation response index, aggregate index of systemic inflammation, and modified Glasgow prognostic score) were calculated. Exacerbation frequency was defined as the annualized number of moderate-to-severe exacerbations. Patients were classified according to Global Initiative for Chronic Obstructive Lung Disease groups A, B, and E, and associations between inflammatory biomarkers and clinical outcomes were evaluated using appropriate statistical methods, including receiver operating characteristic curve analyses.

**RESULTS:**

Elevated levels of neutrophil-to-lymphocyte ratio, platelet-to-lymphocyte ratio, systemic immune-inflammation index, aggregate index of systemic inflammation, modified Glasgow prognostic score, and C-reactive protein were significantly associated with increased exacerbation frequency and hospitalization rates in the overall cohort (all p<0.05), whereas systemic inflammation response index showed no significant association. Receiver operating characteristic analyses demonstrated moderate discriminative ability of C-reactive protein and inflammatory indices for predicting frequent exacerbations.

**CONCLUSION:**

Systemic inflammatory biomarkers were associated with adverse clinical outcomes in patients with chronic obstructive pulmonary disease and were significantly related to exacerbation frequency and severity. These findings suggest that systemic inflammatory markers may be useful for overall clinical assessment and identifying patients at increased risk of exacerbations, rather than for stage-specific risk stratification.

## INTRODUCTION

Chronic obstructive pulmonary disease (COPD) is a chronic respiratory disease characterized by progressive airflow limitation in the lungs, and it’s one of the leading causes of morbidity and mortality worldwide. Traditionally, the pathophysiology of COPD was thought to be limited to an inflammatory response against harmful particles or gases, such as those from chronic cigarette smoke exposure. However, recent studies have shown that this inflammatory process is not confined to the lungs but also affects systemic circulation, which in turn negatively impacts the course and prognosis of COPD^1,2^.

Systemic inflammation is associated with important clinical outcomes of COPD, such as exacerbation frequency, disease severity, and comorbidities. This suggests that inflammation is not just a biological response but a fundamental mechanism that accelerates disease progression and severely reduces patients’ quality of life. Therefore, identifying systemic inflammation, even in the stable phase of COPD, can play a significant role in predicting patients’ future risk of exacerbation and overall survival^3^.

Various systemic inflammation biomarkers, particularly ratios that are easily obtained from a complete blood count and provide information about the severity of inflammation, have recently gained significant interest in clinical practice. Some of these biomarkers include the neutrophil-to-lymphocyte Ratio (NLR), platelet-to-lymphocyte ratio (PLR), systemic immune-inflammation index (SII), systemic inflammation response index (SIRI), aggregate index of systemic inflammation (AISI), and modified Glasgow prognostic score (mGPS). It is known that these biomarkers have the potential to assess prognosis and disease severity in various chronic diseases. However, their prognostic value in COPD patients, especially regarding future exacerbation frequency and long-term survival, has not been fully elucidated^4^.

This study aims to investigate the prognostic value of systemic inflammation biomarkers, measured during the stable phase, on disease severity, exacerbation frequency, and overall survival in patients diagnosed with COPD.

## METHODS

### Study design and population

A priori power analysis was performed before study initiation, and the required minimum sample size was calculated as 64 patients. This retrospective cohort study was conducted at a single tertiary training and research hospital and included 132 adult patients (≥18 years) with a confirmed diagnosis of COPD who met the Global Initiative for Chronic Obstructive Lung Disease (GOLD) criteria and were followed in the outpatient clinic for at least 24 months. Blood samples and spirometry were obtained during routine visits when patients were clinically stable, defined as no acute exacerbation, hospitalization, or respiratory infection within the preceding 4 weeks.

Patients with conditions that could significantly affect systemic inflammatory markers (e.g., active infection, malignancy, hematologic, or chronic inflammatory diseases) or with incomplete laboratory or spirometric data were excluded. The study was approved by the institutional ethics committee and conducted in accordance with the Declaration of Helsinki.

### Data collection and baseline assessments

Demographic and clinical data were retrospectively obtained from electronic medical records at study entry, including age, sex, smoking status, comorbidities, and COPD maintenance therapies (inhaled corticosteroids, long-acting β_2_-agonists, and long-acting muscarinic antagonists). Pulmonary function test parameters—forced expiratory volume in one second (FEV_1_), forced vital capacity (FVC), and the FEV_1_/FVC ratio—were recorded from spirometry performed during routine outpatient visits in the clinically stable state closest to the time of blood sampling. Patients were classified according to Global Initiative for Chronic Obstructive Lung Disease (GOLD) stages based on post-bronchodilator FEV_1_ values.

### Laboratory measurements and inflammatory indices

Baseline laboratory data were obtained from venous blood samples collected during clinically stable outpatient visits and included complete blood count parameters (neutrophils, lymphocytes, monocytes, and platelets), C-reactive protein (CRP), and serum albumin levels. To minimize bias related to transient inflammatory conditions, medical records were reviewed to exclude concurrent infections or other acute illnesses at the time of sampling.

### Systemic inflammatory indices were calculated using standard formulas

Systemic inflammatory indices were calculated using standard formulas: NLR (neutrophil count/lymphocyte count), PLR (platelet count/lymphocyte count), SII (platelet count×neutrophil count/lymphocyte count), SIRI (neutrophil count×monocyte count/lymphocyte count), AISI (neutrophil count×monocyte count×platelet count/lymphocyte count), and the mGPS. The mGPS was defined as follows: score 0, CRP≤10 mg/L and albumin ≥35 g/L; score 1, CRP>10 mg/L and albumin ≥35 g/L; and score 2, CRP>10 mg/L and albumin <35 g/L^5,6^.

### Clinical outcomes and follow-up

Clinical outcomes were evaluated over a fixed 24-month follow-up period after baseline assessment. The primary outcome was the annualized COPD exacerbation frequency, defined as the total number of moderate-to-severe exacerbations divided by the duration of follow-up in years. Moderate exacerbations required outpatient treatment with antibiotics and/or systemic corticosteroids, while severe exacerbations required hospitalization. Secondary outcomes included exacerbation-related hospitalization rates, total length of hospital stay, and all-cause mortality. In patients who died during follow-up, exacerbation rates were adjusted according to the available observation period to minimize underestimation.

### Ethical declaration

Our institution has granted ethics committee approval with protocol number 2871/14.01.2025 As this was retrospective research, no informed consent was obtained from participants.

### Statistical analysis

Statistical analyses were performed using International Business Machines Statistical Package for the Social Sciences Statistics for macOS, version 30.0 (IBM Corp., Armonk, NY). Categorical variables were summarized as frequencies and percentages, and continuous variables as mean±standard deviation or median (minimum–maximum), as appropriate. Normality was assessed using the Kolmogorov-Smirnov test. Intergroup comparisons were performed using the Kruskal-Wallis test for continuous variables and the chi-square or Fisher’s exact test for categorical variables, with Bonferroni correction applied for post hoc analyses. Associations between continuous variables were evaluated using Spearman’s correlation analysis. Receiver operating characteristic (ROC) curve analyses were conducted to assess the predictive performance of systemic inflammatory biomarkers for frequent exacerbations, with area under the curve (AUC) values and optimal cut-off points calculated where appropriate. Statistical significance was defined as a two-sided p<0.05.

## RESULTS

The demographic and clinical characteristics of the 132 patients stratified according to GOLD groups are presented in Table 1. The study population was predominantly elderly (mean age 72.0±7.0 years), male (75.8%), and ex-smokers (79.5%), with moderate-to-severe airflow limitation (mean FEV_1_ 43.2% predicted; FEV_1_/FVC 61.8%). A history of prior exacerbations was present in 81.8% of patients, with a mean of 2.3 exacerbation events during follow-up.

**Table 1:** Distribution of patients’ demographic and clinical findings according to Global Initiative for Chronic Obstructive Lung Disease stages.

Variables (n=132)	Gold-A (n=18)	Gold-B (n=45)	Gold-E (n=69)	p-value	Post-hoc^*^
	n (%) or median (min–max)	n (%) or median (min–max)	n (%) or median (min–max)		
Age (years)	70 (65–82)	68 (65–85)	71 (65–96)	0.179	
Sex n (%)				0.012	
Female	**2 (11.1)**	**6 (13.3)**	**24 (34.8)**		
Male	**16 (88.9)**	**39 (86.7)**	**45 (65.2)**		
BMI	26.7 (19–35.7)	26.1 (15.6–44.4)	25.5 (17.3–37.1)	0.474	
Smoking status				0.073	
Smoker	6 (33.3)	12 (26.7)	9 (13)		
Ex-smoker	12 (66.7)	33 (73.3)	60 (87)		
PFT findings					
FEV1 (%)	**60 (46–79)**	**49 (35–75)**	**34 (13–66)**	**<0.001**	**A–B; A–E; B–E**
FEV1/FVC (%)	72 (44–94)	69 (48–96)	72 (38–100)	0.665	
Exacerbation	**8 (44.4)**	**31 (68.9)**	**69 (100)**	**<0.001**	
Frequency of exacerbations	**0 (0–2)**	**1 (0–1)**	**3 (1–9)**	**<0.001**	**A–E; B–E**
Hospitalization	**0 (0)**	**0 (0)**	**41 (59.4)**	**<0.001**	
Number of hospitalizations	**0 (0–0)**	**0 (0–0)**	**1 (0–4)**	**<0.001**	**A–E; B–E**
Laboratory values					
Albumin (g/L)	**41.1 (34.6–45.9)**	**39.3 (29.4–53.6)**	**35.9 (27.9–44.4)**	**<0.001**	**A–E; B–E**
C-reactive protein (mg/L)	12.7 (0.9–55.7)	13.8 (0.8–32.6)	10.5 (1.1–72)	0.492	
White blood cell (×109/L)	7.4 (5.1–10.4)	8.3 (5–10.5)	8.3 (4.1–11)	0.316	
Hemoglobin (g/L)	13.2 (8.7–15.2)	13.7 (8.9–16.6)	13.2 (8.4–17)	0.328	
Hematocrit (%)	39.8 (26.7–45)	41.3 (27–50.2)	40.1 (26.5–49.6)	0.467	
Platelet (×109/L)	245.5 (108–485)	236 (108–337)	243 (117–821)	0.068	
Lymphocyte (×109/L)	1.8 (0.9–2.9)	1.7 (0.6–3.7)	1.7 (0.5–3.6)	0.459	
Neutrophil (×109/L)	4.7 (3.1–8.5)	5.2 (3–8.9)	5.8 (1.9–9.1)	0.125	
Monocyte (×109/L)	0.5 (0.2–1.2)	0.6 (0.1–3.8)	0.5 (0.3–1.4)	0.107	
Inflammation index					
NLR	2.7 (1.5–8.7)	2.7 (1.1–15.6)	3.7 (1.1–17.8)	0.065	
PLR	**137.4 (37.9–408.2)**	**131.3 (37.9–591.2)**	**163.8 (64.5–732.1)**	**0.015**	**B–E**
SII	**698.4 (184.5–3,453.8)**	**615 (184.5–5,273.8)**	**931.7 (221.3–5,930.4)**	**0.009**	**B–E**
SIRI	1.5 (0.6–4.5)	1.6 (0.5–14.8)	1.9 (0.3–10.5)	0.286	
AISI	**338 (126.5–1,796)**	**330.3 (123.1–4,166.3)**	**510.2 (76.1–3,321)**	**0.050**	**B–E**
mGPS				0.430	
0	7 (38.9)	13 (28.9)	32 (46.4)		
1	6 (33.3)	16 (35.6)	21 (30.4)		
2	5 (27.8)	16 (35.6)	16 (23.2)		

BMI: body mass index; NLR: neutrophil-to-lymphocyte ratio; PLR: platelet-to-lymphocyte ratio; SII: systemic immune-inflammation index; SIRI: systemic inflammation response index; AISI: aggregate index of systemic inflammation; mGPS: modified Glasgow prognostic score; PFT: Pulmonary Function Test(s); FEV1: forced expiratory volume in 1 second; FVC: forced vital capacity. Bold values represent statistically significant differences (p<0.05).

*indicates statistically significant results (p<0.05).

Disease severity, symptom burden, exacerbation frequency, hospitalization rates, and systemic inflammatory indices increased progressively from GOLD Group A to Group E (all p<0.001), with Group E showing the most severe airflow limitation and the highest inflammatory marker levels. ROC analysis demonstrated that PLR, SII, and AISI significantly predicted exacerbations (all p<0.05), with PLR showing the best discriminative performance (AUC=0.691; cut-off >138.89, sensitivity 59.3%, specificity 79.2%). SII and AISI showed moderate discriminative ability for predicting exacerbations (AUC=0.636 and 0.630, respectively), with relatively high specificity. In contrast, NLR and SIRI were not statistically significant predictors; despite high sensitivity, NLR’s low specificity limited its standalone utility (Table 2).

**Table 2:** Receiver operating characteristic analysis results of inflammatory biomarkers in predicting exacerbation development.

Variable	AUC (95%CI)	p-value	Cut-off	Sensitivity (%)	Specificity (%)
NLR	0.600 (0.469–0.730)	0.134	>1.9986	85.2	37.5
PLR	0.691 (0.528–0.791)	0.017	>138.89	59.3	79.2
SII	0.636 (0.510–0.763)	0.035	>810.16	49.1	79.2
SIRI	0.563 (0.431–0.694)	0.348	>1.41	69.4	54.2
AISI	0.630 (0.509–0.750)	0.035	>501.93	47.2	83.3

NLR: neutrophil-to-lymphocyte ratio; PLR: platelet-to-lymphocyte ratio; SII: systemic immune-inflammation index; SIRI: systemic inflammation response index; AISI: aggregate index of systemic inflammation; CI: confidence interval; AUC: area under the curve.

Correlation analyses (Table 3) demonstrated significant positive associations between exacerbation frequency and NLR, PLR, SII, and AISI, as well as between hospitalization rates and the same biomarkers (all p<0.01). SIRI was not significantly associated with exacerbation frequency and showed only a marginal association with hospitalizations. No significant correlations were observed within individual GOLD subgroups, likely reflecting limited sample size and event numbers.

**Table 3:** Distribution of the relationship between exacerbation and hospitalization frequency and biomarkers.

Biomarkers		All patients		Gold-A		Gold-B		Gold-E	
		Exacerbation frequency	Hospital admissions	Exacerbation frequency	Hospital admissions	Exacerbation frequency	Hospital admissions	Exacerbation frequency	Hospital admissions
NLR	r	**0.218**	**0.248**	-0.136	–	0.174	–	0.065	0.199
	p	**0.012**	**0.004**	0.590	–	0.254	–	0.594	0.101
	n	**132**	**132**	18	18	45	45	69	69
PLR	r	**0.256**	**0.232**	0.102		0.277		0.025	0.155
	p	**0.003**	**0.008**	0.687	–	0.065	–	0.836	0.202
	n	**132**	**132**	18	18	45	45	69	69
SII	r	**0.278**	**0.273**	-0.115		0.203		0.071	0.176
	p	**0.001**	**0.002**	0.649	–	0.180	–	0.563	0.149
	n	**132**	**132**	18	18	45	45	69	69
SIRI	r	0.143	**0.171**	-0.195	–	0.118	–	0.097	0.157
	p	0.103	**0.050**	0.438		0.439		0.426	0.198
	n	132	**132**	18	18	45	45	69	69
AISI	r	**0.242**	**0.230**	-0.055	–	0.170	–	0.098	0.156
	p	**0.005**	**0.008**	0.828		0.264		0.425	0.201
	n	**132**	**132**	18	18	45	45	69	69
mGPS	r	-0.138	-0.081	0.168	–	-0.130	–	0.003	0.037
	p	0.116	0.356	0.505	–	0.396	–	0.980	0.764
	n	132	132	18	18	45	45	69	69

NLR: neutrophil-to-lymphocyte ratio; PLR: platelet-to-lymphocyte ratio; SII: systemic immune-inflammation index; SIRI: systemic inflammation response index; AISI: aggregate index of systemic inflammation; mGPS: modified Glasgow prognostic score. Bold values represent statistically significant differences (p<0.05).

## DISCUSSION

This retrospective cohort study aimed to investigate the potential of systemic inflammatory markers—specifically NLR, PLR, SII, SIRI, AISI, and mGPS—to predict future exacerbation frequency, hospitalizations, and all-cause mortality in stable patients with COPD. Our findings demonstrate that NLR, PLR, SII, and AISI are significantly associated with a higher number of both exacerbations and hospitalizations in the overall study population. These results support the established understanding that systemic inflammation, a key component of COPD pathogenesis, plays a crucial role in determining the clinical progression and prognosis of the disease^7,8^.

Numerous studies in the literature have examined the relationship between inflammatory markers and COPD exacerbations. For instance, some research has established that NLR and PLR are independent predictors of exacerbation frequency and mortality in COPD patients^9,10^. Our findings support and strengthen this evidence. Furthermore, our demonstration that next-generation inflammatory markers like SII and AISI are also significantly associated with exacerbations and hospitalizations is a crucial contribution. This finding supports the potential use of these simple and inexpensive tests as prognostic tools in routine clinical practice.

A notable finding in our study is that while systemic inflammatory markers showed a significant correlation with exacerbations and hospitalizations in the overall patient cohort, these correlations were not statistically significant within the individual GOLD subgroups (A, B, and E). This result suggests that the GOLD classification system, which primarily relies on airflow limitation and a patient’s symptom and exacerbation history, may not fully capture or reflect the underlying inflammatory status of each subgroup. Consequently, this indicates that inflammatory markers might serve as a valuable supplement to the GOLD classification in a broader patient population. However, their predictive ability may be limited when applied to the specific, less severe characteristics of each distinct subgroup^11^.

In a prospective study, the role of NLR and PLR in predicting frequent exacerbations in COPD was investigated. Blood parameters of patients in the stable phase were regularly monitored, and the values were compared when an exacerbation occurred. In patients prone to exacerbations, NLR and PLR values were found to be statistically significantly higher in the pre-exacerbation period compared to patients who did not experience an exacerbation. This suggests that these markers may reflect the pre-exacerbation inflammatory state^12^.

A retrospective study examined the relationship between SII values and disease severity in stable COPD patients. It was found that SII values were significantly higher in patients with a more advanced GOLD stage compared to those with a lower stage. SII showed a positive correlation with traditional severity markers in COPD, such as the degree of airflow limitation (FEV1%) and symptom scores. This finding suggests that SII may reflect not only the risk of exacerbation but also the underlying severity of the disease^13^.

A study showed that a high mGPS score is associated with poor clinical outcomes (the need for intensive care unit transfer and in-hospital mortality) in patients with Acute Exacerbation of Chronic Obstructive Pulmonary Disease (AE-COPD). This study investigated the value of mGPS in predicting mortality in patients hospitalized for acute COPD exacerbation. Specifically, patients with a GPS score of 2 were found to have a higher risk of adverse outcomes compared to those with lower scores. This suggests that mGPS may be an independent risk factor for mortality in AE-COPD^14^.

One of the main strengths of our study is that it examined not only classic markers (NLR and PLR) but also newer and more comprehensive markers like SII and AISI. Additionally, the detailed examination of the followed patient group and clinical outcomes increases the reliability of our findings. Several contemporary studies have reported associations between bloodbased inflammatory markers—particularly NLR and PLR—and exacerbation frequency, hospitalization, and mortality in COPD^15^. Composite indices such as SII and AISI, which integrate multiple immune cell populations, have recently gained attention as more comprehensive markers of systemic inflammation and immune dysregulation^16^. The observed associations in our study support the biological plausibility that these indices may capture the cumulative inflammatory burden more effectively than single biomarkers.

### Limitations

This study has several limitations. Its retrospective, single-center design limits causal inference and generalizability. Although blood samples were obtained during clinically stable periods and patients with recent exacerbations or infections were excluded, subclinical inflammation at the time of sampling cannot be fully ruled out. Residual confounding from unmeasured inflammatory conditions may also have influenced biomarker levels. In addition, no a priori power analysis was performed, and subgroup analyses may have been underpowered. Detailed longitudinal data on treatment changes, adherence, and the use of add-on therapies were incomplete. Finally, although mortality was considered in exacerbation rate calculations, competing risks related to death may still have affected the results. Despite these limitations, the study provides clinically relevant real-world data on readily available inflammatory biomarkers in COPD.

## CONCLUSION

Systemic inflammatory biomarkers measured during stable COPD were associated with future exacerbation burden, with higher baseline CRP and composite indices linked to more frequent moderate-to-severe exacerbations. Although causality cannot be inferred due to the retrospective design, these readily available biomarkers may serve as practical adjuncts for risk stratification in routine clinical practice. Prospective multicenter studies are needed to validate these findings and clarify their role in personalized COPD management.

## Data Availability

The datasets generated and/or analyzed during the current study are available from the corresponding author upon reasonable request.
